# Ferroelectric‐Polarization‐Driven Structural Engineering of Bi_3_Nb_17_O_47_ Anodes for High‐Performance Lithium‐Ion Batteries

**DOI:** 10.1002/advs.202520293

**Published:** 2026-01-22

**Authors:** Xiaoming Lou, Songjie Li, Chunfu Lin, Yanchao Li, Mingze Sun, Zhen Liu, Weiye Nie, Xiaolong Tang, Baoming Wang, Wenwu Cao, Renchao Che

**Affiliations:** ^1^ College of Physics Donghua University Shanghai China; ^2^ Center of Acoustic Functional Materials and Applications School of Materials Science and Intelligent Engineering Nanjing University Suzhou China; ^3^ Laboratory of Advanced Materials Shanghai Key Lab of Molecular Catalysis and Innovative Materials State Key Laboratory of Coatings for Advanced Equipment College of Smart Materials and Future Energy Fudan University Shanghai China; ^4^ Institute of Materials for Energy and Environment School of Materials Science and Engineering Qingdao University Qingdao China; ^5^ School of Materials Science and Engineering Nanjing University of Science and Technology Nanjing Jiangsu China

**Keywords:** electrochemical energy‐storage material, ferroelectric, niobate, structural engineering, tungsten bronze

## Abstract

Metal‐ion batteries face challenges in optimizing electrode materials to enhance capacities and structural stability. Traditional strategies like defect engineering and elemental doping show limitations, necessitating innovative approaches. Herein, we propose a ferroelectric‐polarization strategy to modulate the crystal structure of Bi_3_Nb_17_O_47_, a tungsten bronze (TTB)‐type anode material with a theoretical capacity of 308 mAh g^−1^ but restricted Li^+^ storage sites. The polarization induces asymmetric displacements of Nb^5+^ and inward contraction of O^2−^, enlarging the Li^+^‐storage cavities by approximately 8%. This structural tailoring significantly boosts the reversible capacities of Bi_3_Nb_17_O_47_ by 36%−55% across various current densities (0.1−10 C). Furthermore, the enlarged Li^+^‐storage cavities enable smaller unit‐cell‐volume fluctuations, resulting in its enhanced cycling stability (84.9% capacity retention after 1000 cycles at 5 C). This work pioneers electric‐field‐driven structural engineering in electrochemical energy‐storage materials, offering a transformative modification strategy for high‐performance metal‐ion batteries.

## Graphical abstract

A transformative ferroelectric‐polarization strategy is employed to overcome the intrinsic limitations of tungsten bronze (TTB)‐type anode materials in metal‐ion batteries, using Bi_3_Nb_17_O_47_ as a model system. By exploiting the non‐centrosymmetric crystal structure and field‐induced ionic displacements, controlled structural engineering with approximately 8% enlarged Li^+^‐storage cavities has been achieved in Bi_3_Nb_17_O_47_, which simultaneously enhances its Li^+^‐storage capability and cycling stability.

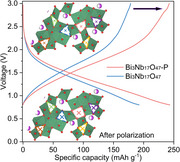

## 1 Introduction

Metal‐ion batteries are highly favored electrochemical energy‐storage systems widely employed in portable electronic devices and electric vehicles [1, 2, 3, 4]. The electrochemical performance of these batteries is fundamentally governed by the crystal structure of energy‐storage materials, where reversible metal‐ion intercalation/deintercalation processes occur [5, 6, 7, 8]. Consequently, structural modifications of energy‐storage materials have emerged as critical strategies for electrochemical‐performance optimization. Taking lithium‐ion batteries (LIBs) as a prime example, conventional approaches primarily focus on defect engineering (e.g., oxygen‐vacancy creation and dislocation generation) and elemental doping [9, 10, 11, 12, 13]. Xia et al. demonstrated that constructing heterostructures in LiMnO_2_ effectively enhances its structural integrity during electrochemical cycling [9]. Li et al. reported that Ti–Mg–Al co‐doping significantly improved the high‐voltage cycling stability of LiCoO_2_ [10]. While these methods have achieved notable progress, developing innovative structural modulation techniques remains imperative to further advance LIBs performance.

Ferroelectric materials, characterized by non‐centrosymmetric crystal structures, exhibit polarization‐dependent ionic displacements under external electric fields. Such field‐induced ion migrations can induce substantial structural modifications, including phase transitions and domain reorientations [14, 15, 16, 17]. For instance, in lead zirconate titanate (PZT), the migrations of Pb^2+^ under polarization can lead to domain wall motion and a transformation from a tetragonal to a rhombohedral phase [18]. In barium titanate (BaTiO_3_), the migrations of Ti^4+^ can cause changes in ferroelectric phase transitions, impacting dielectric and piezoelectric properties [19]. Therefore, integrating ferroelectric properties into energy‐storage materials could enable electric‐field‐controlled structural engineering, offering a promising avenue to enhance electrochemical performance. Recently, the intersection of ferroelectricity and electrochemistry has emerged as a vibrant frontier for high‐performance batteries. Beyond traditional dielectric applications, the intrinsic spontaneous polarization in ferroelectrics can induce a strong internal electric field. This built‐in field has been theoretically and experimentally proven to lower the activation energy for metal‐ion diffusion, modulate the band structure at the electrode/electrolyte interface, and mitigate space‐charge layers [20, 21].

Herein, we focus on tungsten bronze (TTB)‐type oxides, a class of intercalation anodes distinguished by their open tunnel structures and superior safety performance. Unlike commercial graphite which suffers from potential safety hazards (Li plating), and Li_4_Ti_5_O_12_ (LTO) which is strictly limited by its low theoretical capacity (∼175 mAh g^−1^), TTB materials offer a compelling advantage by combining high safe performance with much higher theoretical capacities. The TTB family features a framework of corner‐sharing MO_6_ octahedra, forming pentagonal, square, and triangular tunnels. A distinguishing feature of TTB materials is their rich phase transition behavior. The flexible rotation and tilting of the MO_6_ octahedra often lead to various polymorphic phase transitions and structural distortions under external stimuli (e.g., temperature, electric field, or ionic insertion). While this structural flexibility offers potential for ion intercalation, it also renders the material susceptible to irreversible phase changes and lattice strain during cycling. Therefore, precisely controlling these structural distortions via an external field can offer a novel pathway to stabilize the framework while expanding the storage channels [22, 23, 24, 25, 26].

In this work, we propose a pioneering strategy to improve electrochemical performance through ferroelectric‐polarization engineering, employing the corona poling technique (CPT). Our investigation identifies Bi_3_Nb_17_O_47_ within the M–Nb–O system as a representative TTB candidate with a theoretical capacity of 308 mAh g^−1^ (significantly higher than that of LTO), yet constrained by the aforementioned insufficient Li^+^‐storage sites. The polarization induces coordinated Nb^5+^‐cation displacements from octahedral centers, coupled with O^2−^ contraction via Nb─O bond interaction. This structural rearrangement expands the total cavity volume for Li^+^ storage by approximately 8%, yielding remarkable capacity enhancement of 36%, 42%, 48%, 51%, 52%, and 55% at 0.1, 0.5, 1, 2, 5, and 10 C, respectively. In situ X‐ray diffraction (XRD) analyses demonstrate that polarized Bi_3_Nb_17_O_47_ (Bi_3_Nb_17_O_47_‐P) exhibits attenuated unit‐cell‐volume fluctuations (Δ*V* = 0.0619% per Li^+^ insertion vs. 0.0842% in pristine) due to the enlarged Li^+^‐storage sites, correlating with improved long‐term stability (84.9% capacity retention after 1000 cycles at 5C). This strategy concurrently resolves dual challenges of capacity limitations and structural degradation in TTB anodes, establishing an electric‐field‐driven paradigm for next‐generation energy‐storage materials.

## 2 Results and Discussion

### 2.1 Ferroelectric and Dielectric Properties of Bi_3_Nb_17_O_47_

**FIGURE 1:**
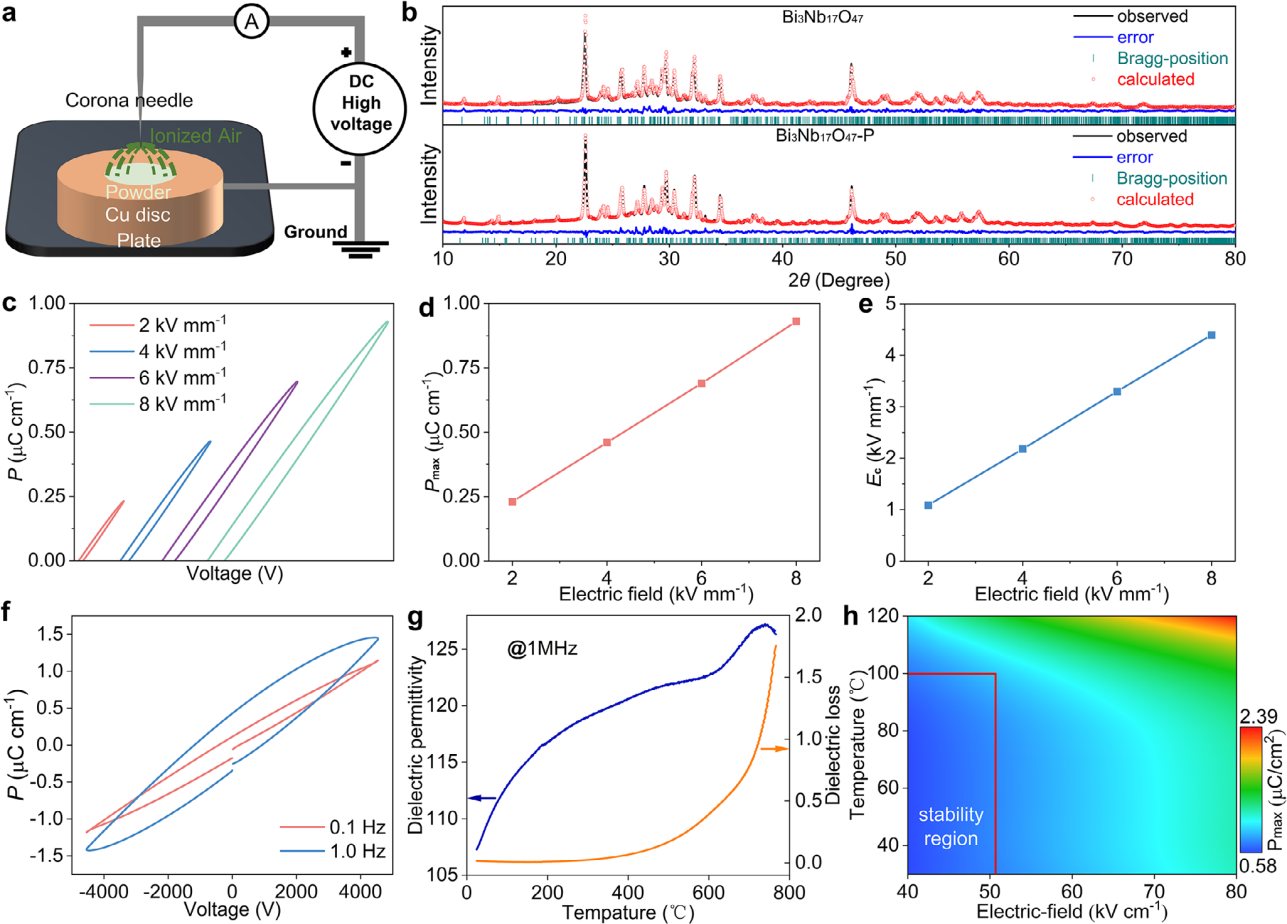
Crystal‐structure evolutions, ferroelectric and dielectric performance of Bi_3_Nb_17_O_47_. (a) Schematic diagram of the corona poling technique. (b) Rietveld‐refined XRD patterns. (c) Electric fields (2, 4, 6, and 8 kV mm^−1^) dependence of hysteresis loops. (d) Relationship between max polarization value (*P*_max_) and electric field. (e) Relationship between coercive field (*E*_c_) and electric field. (f) Hysteresis loops at 0.1 and 1 Hz. (g) Dielectric constant and loss vs. temperature curves of Bi_3_Nb_17_O_47_. (h) Counter of *P*_max_ depending on temperature and electric field.

Bi_3_Nb_17_O_47_‐P powders were obtained using CPT (Figure 1a). The XRD analyses (Figure S1) and Rietveld refinements (Figure 1b) confirm phase purity of Bi_3_Nb_17_O_47_ and Bi_3_Nb_17_O_47_‐P powders through JCPDS 25–0091 pattern matching. Bi_3_Nb_17_O_47_ belongs to a family of A*_x_* (MO)*_y_* MO_3_ (*x* = 1/5, *y* = 2/15, A = Bi, M = Nb) compounds, based on a bronze‐type MO_3_ network, where certain tunnels are occupied by MO species, while others are filled with cations A, resulting in a non‐centrosymmetric structure capable of ferroelectric polarization. The ferroelectric origin lies in the cooperative off‐centering displacements of *d* ^0^ Nb^5+^ cations within the oxygen octahedra driven by the second‐order Jahn–Teller (SOJT) effect, combined with the stereochemical activity of the Bi^3+^ 6*s* ^2^ lone pairs, which collectively break the inversion symmetry [27, 28, 29]. The complementary XRD analysis of bulk Bi_3_Nb_17_O_47_ ceramics (Figure S2) confirms phase consistency with the powder sample, indicating the pure phase of Bi_3_Nb_17_O_47_ ceramics.

Figure 1c shows the polarization−electric field (P−E) hysteresis loops of Bi_3_Nb_17_O_47_ ceramics measured under varying electric fields (2−8 kV mm^−1^). The hysteresis loop area expands progressively with increasing field strength, accompanied by enhanced tilt angles relative to the electric field (*E*) axis. Both maximum polarization (*P*_max_) and coercive field (*E*_c_) exhibit near‐linear increases with applied field (Figure 1d,e), demonstrating field‐dependent ferroelectric response [30, 31]. Frequency‐dependent P−E hysteresis loops acquired at 8 kV mm^−1^ reveal distinct loss characteristics (Figure 1f). These losses originate from Nb^5+^ migrations concurrent with polarization switching under applied fields, a process that modifies local crystal structures. Notably, frequency elevation from 0.1 to 1 Hz induces loop widening, indicative of delayed domain switching dynamics [32, 33]. The thermal stability of polarization is evaluated through temperature‐dependent *P*_max_ measurements (4−8 kV mm^−1^, 1 Hz) across 20−120°C (Figure 1h). Remarkable stability emerges in the 4−5 kV mm^−1^ range, maintaining consistent *P*_max_ values between 20°C−100°C. This operational temperature window aligns with LIBs working conditions (<80°C), confirming practical applicability.

Figure 1g shows the temperature dependence of the dielectric properties of polarized Bi_3_Nb_17_O_47_ ceramics measured at 1 MHz. The dielectric permittivity curve exhibits two dielectric anomaly peaks at around 350°C and 740°C, representing the ferroelectric‐to‐relaxor phase transition and the maximum dielectric constant respectively. It is important to observe that the cement's loss becomes more pronounced at high frequencies, particularly at 1 MHz, where a characteristic oval shape indicative of conduction is evident. This heightened sensitivity is attributed to the fact that, at higher frequencies, interfacial polarization struggles to track fluctuation in the electric field due to the extended time required for space‐charge polarization to develop [34, 35]. Such high dielectric loss and percolation threshold, indicate high electronic conductivity, showing great potential for LIBs practical applications. Moreover, the value of the degree of diffuseness (*γ*, Figure S3) is approximately 1.27, confirming the relaxor‐type ferroelectric behavior of the Bi_3_Nb_17_O_47_ ceramics [36, 37].

### 2.2 Characteristics of Bi_3_Nb_17_O_47_ Before and After Polarization

**FIGURE 2:**
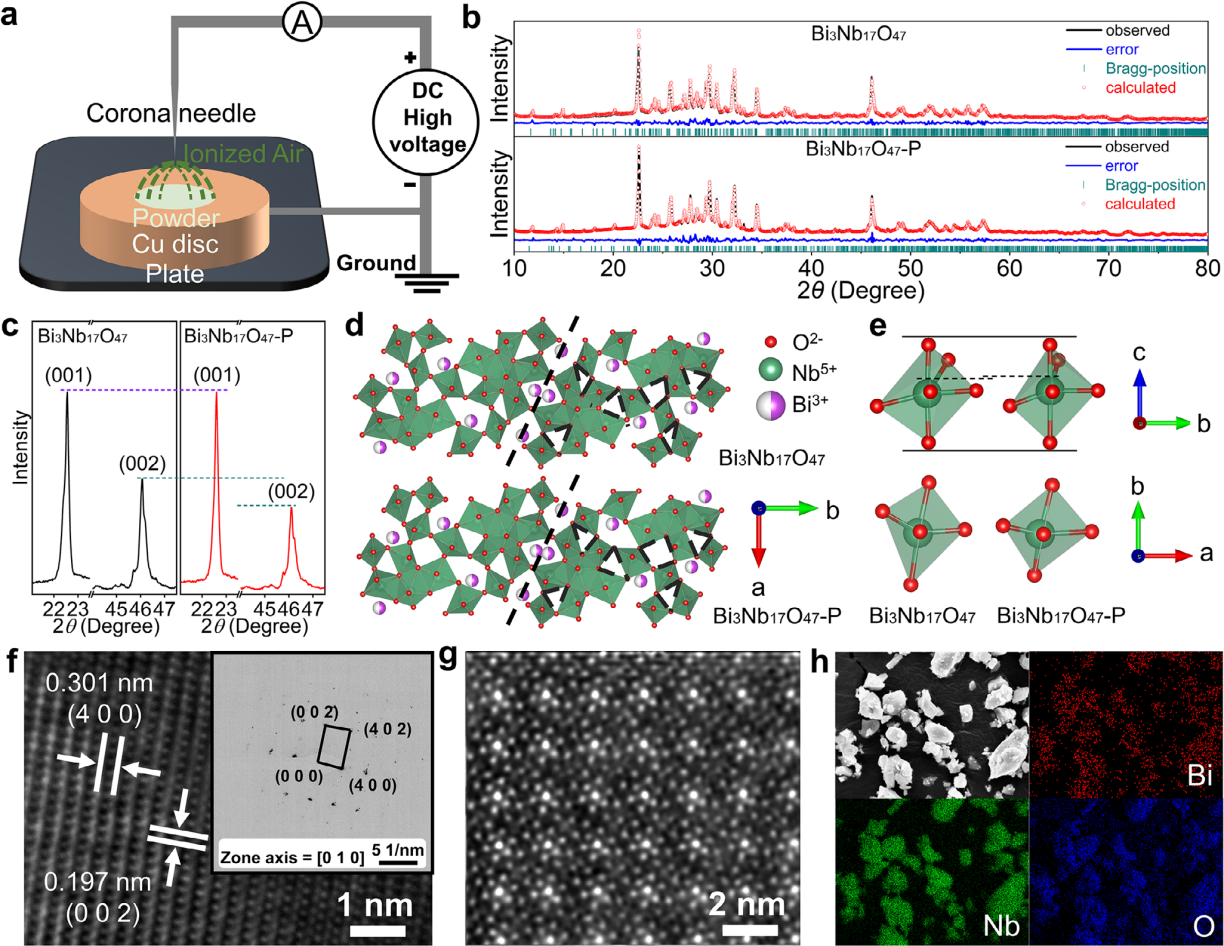
Crystal‐structure evolution and physico‐chemical characterizations of Bi_3_Nb_17_O_47_ before and after polarization. (a) details of (001) and (002) XRD peaks, (b) schematic crystal structures (black dashed lines indicate crystallographic symmetry axes), and (c) octahedral structures of Bi_3_Nb_17_O_47_ and Bi_3_Nb_17_O_47_‐P. Contours of Nb^5+^ positions in (d) Bi_3_Nb_17_O_47_ and e) Bi_3_Nb_17_O_47_‐P. f) FESEM image, (g) EDX mapping images, and (h) HRTEM image of Bi_3_Nb_17_O_47_‐P. (i) HAADF image of the area viewed along the *z*‐axis.

The Rietveld refinements reveal polarization‐induced structural modifications (Figure 1b), with a diminished (002)/(001) peak intensity ratio in Bi_3_Nb_17_O_47_‐P (Figure 2a). The post‐polarization analysis, calculated based on the Rietveld‐refined atomic coordinates using VESTA software, demonstrates an approximately 8% octahedral cavity expansion (Figure 2b). This structural evolution is critical for the enhanced Li^+^ storage for two main reasons. First, thermodynamically, the expanded lattice reduces the steric hindrance and electrostatic repulsion for Li^+^ accommodation, potentially activating “dead” sites that were previously too small or energetically unfavorable for Li^+^ insertion. Second, kinetically, the enlarged lattice provides optimized ion‐migration channels, ensuring that these newly activated sites remain effectively accessible/utilizable during the electrochemical process (as supported by the following sections). As detailed in Figure 2d,e, Nb^5+^ exhibits *z*‐axis displacement from 0.552 (pristine) to 0.566 (polarized), deviating further from the (002) plane centroid. This displacement, facilitated by Nb─O bond‐mediated O^2−^ contraction (Figure 2c), reduces octahedral cross‐sectional area while increasing peripheral cavity volume. Crystallographically, the diffraction intensity is governed by the structure factor (*F*_hkl_), which depends heavily on the atomic positions. The Rietveld refinement reveals that the Nb^5+^ cations exhibit a *z*‐axis displacement from 0.552 (pristine) to 0.566 (polarized), deviating further from the (002) plane centroid. This specific displacement of heavy Nb atoms alters the structure factor phase term, leading to a reduction in the structure factor amplitude (*F*_002_) and explicitly accounting for the attenuated (002) peak intensity. This confirms that the macroscopic peak variation originates from the microscopic ferroelectric off‐centering displacement. The lattice‐parameter evolution (Tables S1 and S2) quantitatively corroborates these modifications.

High‐resolution transmission electron microscopy (HRTEM) images are analyzed to further elucidate the crystal structures of Bi_3_Nb_17_O_47_ and Bi_3_Nb_17_O_47_‐P. The two sets of lattice fringes with *d*‐spacing values of 0.635 and 0.393 nm (Figure S4) correspond to the (210) and (001) crystallographic planes of Bi_3_Nb_17_O_47_, respectively. Similarly, those with *d*‐spacing values of 0.301 and 0.197 nm (Figure 2h) match with the (400) and (002) plane of Bi_3_Nb_17_O_47_‐P. This observation is reinforced by the selected‐area electron diffraction (SAED) patterns of Bi_3_Nb_17_O_47_ and Bi_3_Nb_17_O_47_‐P showed *inset* of Figure 2h and Figure S4, confirming structural integrity with characteristic TTB phase signatures in both materials. Figure 2i shows a high‐angle annular dark‐field (HAADF) *z*‐axis image of Bi_3_Nb_17_O_47_‐P. This material still maintains the TTB structure after polarization. Field‐emission scanning electron microscopy (FESEM) images (Figure 2f; Figure S5a) illustrate comparable block‐like morphologies for both samples, with particle dimensions ranging from 0.5 to 5 µm. These findings indicate that CPT has minimal influences on the particle morphology and size. Moreover, the uniform distributions of Bi, Nb, and O (Figure 2g; Figure S5b) confirm the phase purity and compositional uniformity of both samples. These observations collectively demonstrate that structural modifications rather than morphological changes drive the enhanced electrochemical performance in polarized samples (see the following sections).

### 2.3 Crystal‐Structure Evolution of Bi_3_Nb_17_O_47_ and Bi_3_Nb_17_O_47_‐P During GCD Processes

**FIGURE 3:**
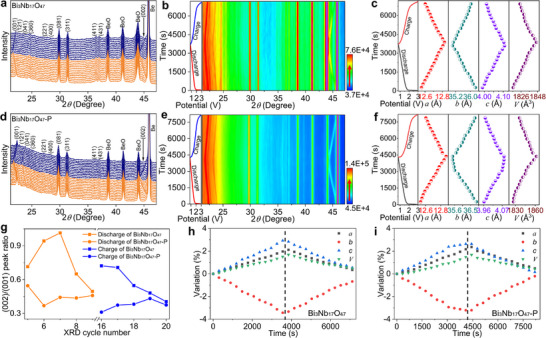
Crystal‐structure evolutions of Bi_3_Nb_17_O_47_ and Bi_3_Nb_17_O_47_‐P. (a) Pristine and (b) contour in situ XRD patterns of Bi_3_Nb_17_O_47_/Li in situ cell with corresponding GCD curves (first cycle). (d) Pristine and (e) contour in situ XRD patterns of Bi_3_Nb_17_O_47_‐P/Li in situ cell with corresponding GCD curves (first cycle). Lattice‐parameter variations of (c,h) Bi_3_Nb_17_O_47_ and (f, i) Bi_3_Nb_17_O_47_‐P during GCD processes. (g) (002)/(001) peak intensity ratio from fifth to nineth cycles (discharge process) and from 16th to 20th cycles (charge process).

During the galvanostatic charge–discharge (GCD) processes at room temperature between 0.8 and 3.0 V, both pristine (Figure 3a,d) and contour (Figure 3b,e) in situ XRD patterns of Bi_3_Nb_17_O_47_/Li and Bi_3_Nb_17_O_47_‐P/Li in situ cells show superior reversibility and stability. When discharge (Li^+^ insertion) from 3.0 to 0.8 V, the (001), (121), (041), (400), and (002) peaks progressively shift toward lower 2*θ* angles, indicating the successful insertion of Li^+^ into Bi_3_Nb_17_O_47_ and Bi_3_Nb_17_O_47_‐P lattice [38, 39]. This process is well‐reversed during the subsequent charge (Li^+^ extraction) process from 0.8 to 3.0 V, suggesting the high first‐cycle Coulombic efficiencies and structure stability of both Bi_3_Nb_17_O_47_ and Bi_3_Nb_17_O_47_‐P.

Due to the (002) peak being located in a narrow space between peaks of BeO and Be during the GCD processes, the (002) peak merges with the Be peak at around 3.0 V and merges with the BeO peak at around 0.8 V, then the data for analyses can be selected from the fifth to nineth cycles (discharge) and the 16th to 20th cycles (charge). According to the XRD results previously discussed, the polarization process can reduce the (002)/(001) peak ratio, indicating the migrations of Nb^5+^. The same phenomenon is observed in in situ XRD results (Figure 3g), as expected. The (002)/(001) peak ratio of Bi_3_Nb_17_O_47_‐P is lower than that of Bi_3_Nb_17_O_47_ at the same depth of discharge of state (DOC) and state of charge (SOC), indicating that the effects of polarization persist throughout the GCD processes. More interestingly, the (002)/(001) peak ratios of the two samples exhibit opposite trends during the GCD processes. During the discharge process (1.35−1.08 V), the (002)/(001) peak ratio of Bi_3_Nb_17_O_47_ first increases and then decreases, while that of Bi_3_Nb_17_O_47_‐P first decreases and then increases. During the charge process (1.56−1.91 V), the (002)/(001) peak ratio of Bi_3_Nb_17_O_47_ obviously decreases, while that of Bi_3_Nb_17_O_47_‐P increases, indicating the complicated migrations of Nb^5+^ during the GCD processes, and the opposite trend may be due to the internal electric‐field changes induced by polarization and differences in initial Nb^5+^ positions.

The quasi‐symmetric variations in the lattice parameters (*a*, *b*, *c*, and *V*) during the GCD processes (Figure 3c,h for Bi_3_Nb_17_O_47_ and Figure 3f,i for Bi_3_Nb_17_O_47_‐P) are identified by calculating the lattice parameters using Bragg's law, and the values of the two samples exhibit identical trends during the GCD processes. During the lithiation process (3.0−0.8 V), the *a*, *c*, and *V* values increase, while the *b* value decreases. The total changes of the *a*, *b*, *c*, and *V* values in Bi_3_Nb_17_O_47_ at 0.8 V are +0.1057, −0.1716, +0.1462, and +0.0842% (vs. the original values), corresponding to the insertion of one Li^+^ per Li*_x_* Bi_3_Nb_17_O_47_, respectively. As a comparison, those in Bi_3_Nb_17_O_47_‐P are +0.0874, −0.1186, +0.0974, and +0.0619% (vs. the original values). The lattice‐parameter changes in Bi_3_Nb_17_O_47_‐P are obviously smaller than those of Bi_3_Nb_17_O_47_, due to the larger total cavity area from the *c*‐axis direction for Li^+^ insertion, indicating a more stable structure of Bi_3_Nb_17_O_47_‐P during the GCD processes.

### 2.4 Electrochemical Performance of Bi_3_Nb_17_O_47_ and Bi_3_Nb_17_O_47_‐P

**FIGURE 4:**
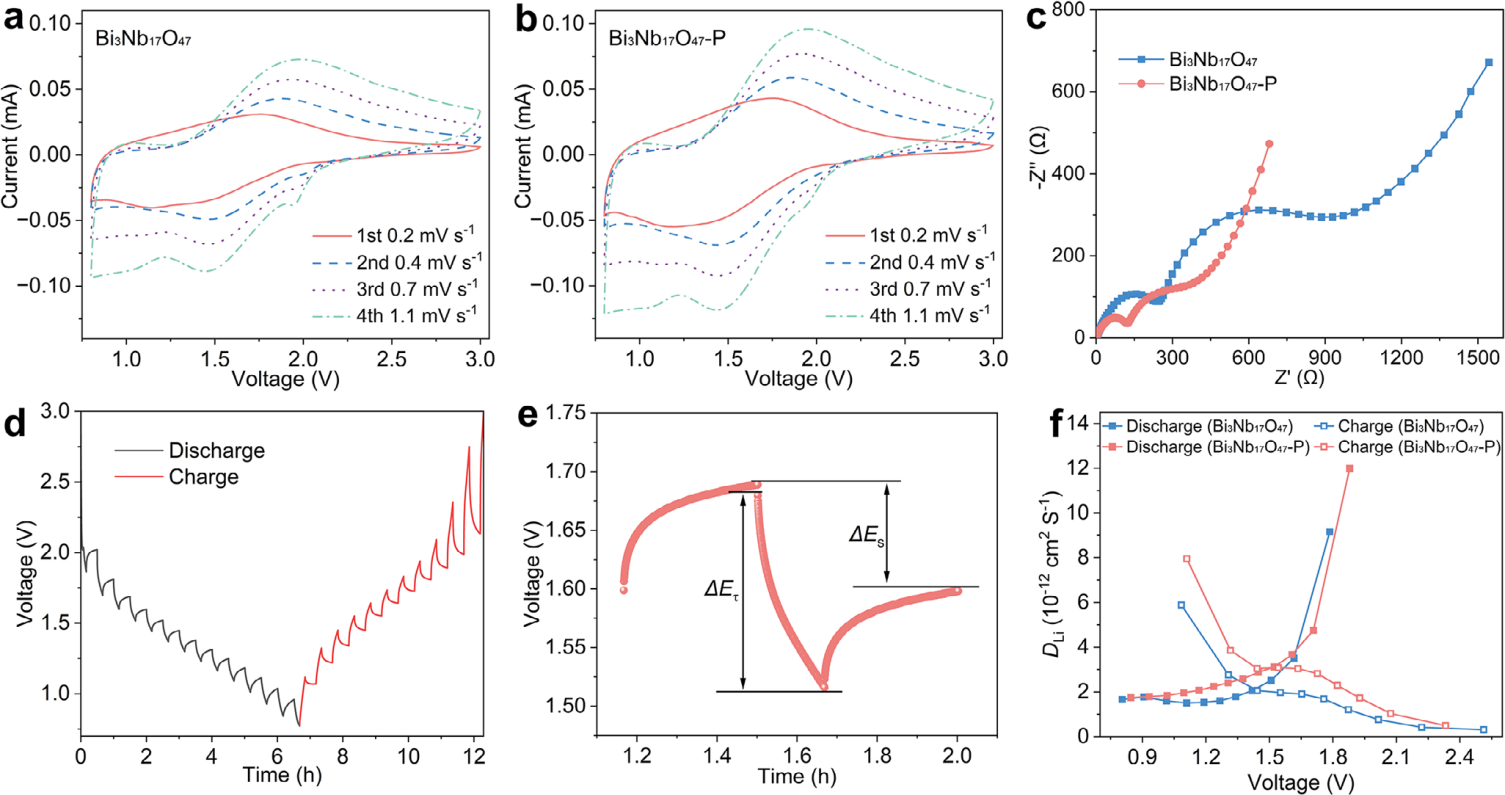
Redox mechanism and electrochemical kinetics of Bi_3_Nb_17_O_47_ and Bi_3_Nb_17_O_47_‐P. CV curves (from 0.2 to 1.1 mV s^−1^) of (a) Bi_3_Nb_17_O_47_ and (b) Bi_3_Nb_17_O_47_‐P. (c) Nyquist plots of Bi_3_Nb_17_O_47_/Li and Bi_3_Nb_17_O_47_‐P/Li half cells. (d) Second‐cycle GITT discharge‒charge profiles of Bi_3_Nb_17_O_47_‐P at 0.5 C. (e) *E* vs. *t* profiles for a single step in the GITT experiment of Bi_3_Nb_17_O_47_‐P at 0.5 C. (f) Li^+^‐diffusion coefficients of Bi_3_Nb_17_O_47_ and Bi_3_Nb_17_O_47_‐P.

The cyclic voltammetry (CV) curves of the Bi_3_Nb_17_O_47_/Li and Bi_3_Nb_17_O_47_‐P/Li half cells at 0.2 mV s^−1^ exhibit a pair of broad redox peaks located at approximately 1.6/1.8 V, correspond to the Nb^4+^/Nb^5+^ and Nb^3+^/Nb^4+^ redox reaction (Figure 4a,b). The Bi_3_Nb_17_O_47_‐P/Li half cell exhibits small electrode polarization, attributed to the faster electrochemical reaction kinetics. Furthermore, the significantly increased peak currents suggest that a larger fraction of active Nb centers participates in the redox reaction of Bi_3_Nb_17_O_47_‐P, thereby contributing to its enhanced reversible capacity.

The galvanostatic intermittent titration technique (GITT) and electrochemical impedance spectroscopy (EIS) measurements quantitatively verify the enhanced kinetics of Bi_3_Nb_17_O_47_‐P. Specifically, the Nyquist plots display a reduced charge‐transfer resistance, while the GITT results show that the bulk Li^+^ diffusivity of Bi_3_Nb_17_O_47_‐P is enhanced (Figure 4c–f; Figure S6). Crucially, these improved kinetics play a vital role in ensuring that the expanded Li^+^‐storage cavities (induced by polarization) remain accessible even at high current densities, thereby maintaining the superior specific capacity across the entire rate‐capability test.

**FIGURE 5:**
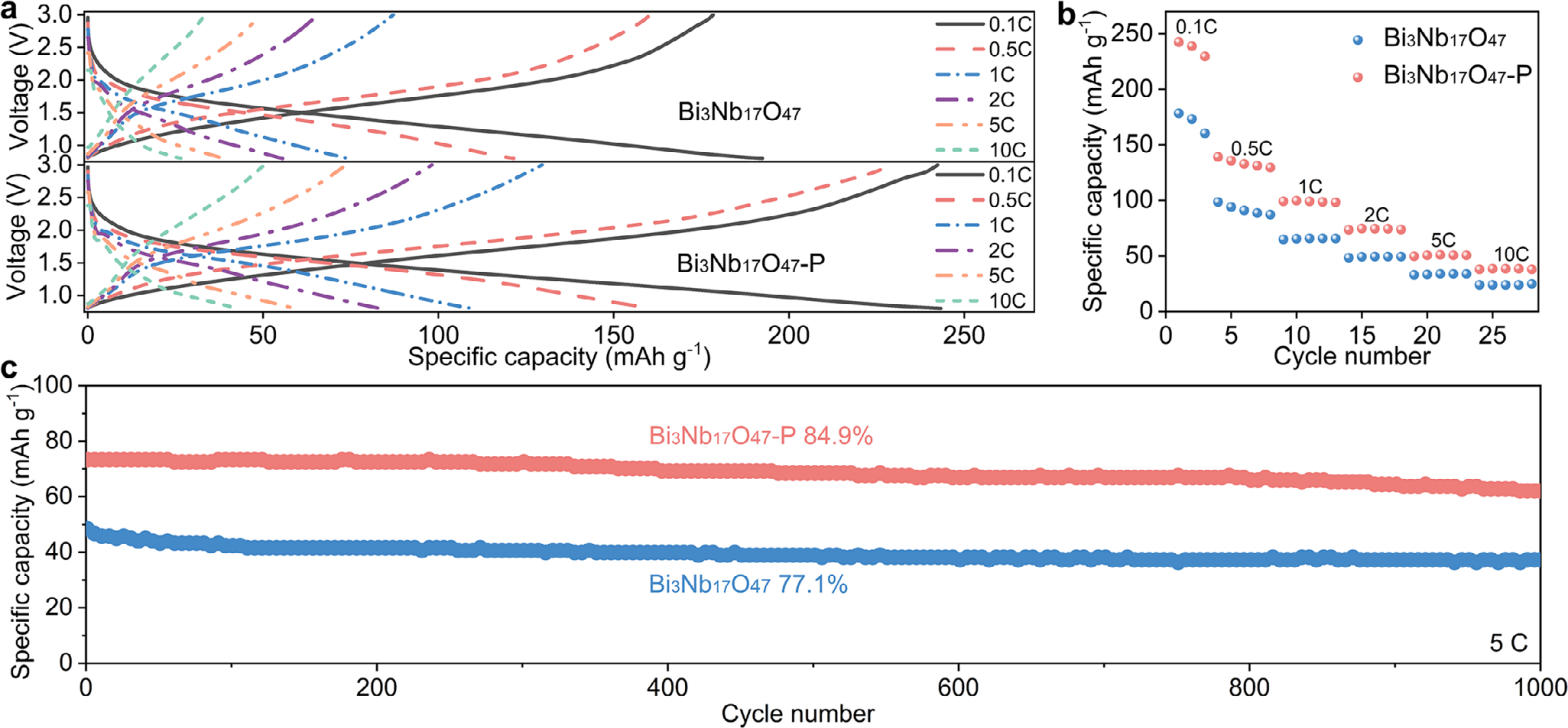
Electrochemical properties of Bi_3_Nb_17_O_47_/Li and Bi_3_Nb_17_O_47_‐P/Li half cells. (a) GCD curves, (b) percentage capacities at 0.1–10 C, and (c) long‐term cycling stability at 5 C.

From the initial two GCD curves of Bi_3_Nb_17_O_47_/Li and Bi_3_Nb_17_O_47_‐P/Li half cells at 25°C between 0.1 and 10 C in the voltage range of 3.0–0.8 V (Figure 5a), it is evident that the voltage profiles before and after polarization exhibit similar shapes, containing a long plateau at ∼1.68 V, which correspond to the Nb^4+^/Nb^5+^ redox couples. Polarized Bi_3_Nb_17_O_47_‐P demonstrates superior Coulombic efficiency (99.6% vs. 92.7% for pristine), ascribed to its stable structure. Moreover, the reversible capacities of Bi_3_Nb_17_O_47_ at 0.1, 0.5, 1, 2, 5, and 10 C are 178, 160, 87, 64, 49, and 33 mAh g^−1^, respectively, while those of Bi_3_Nb_17_O_47_‐P are 242, 228, 129, 97, 74, and 51 mAh g^−1^, respectively (Figure 5b). It is noteworthy that the reversible capacities of Bi_3_Nb_17_O_47_‐P are significantly larger than that of Bi_3_Nb_17_O_47_, corresponding to 36%, 42%, 48%, 51%, 52%, and 55% enhancement at 0.1, 0.5, 1, 2, 5, and 10 C, respectively, which can be attributed to the greater Li^+^‐storage space in Bi_3_Nb_17_O_47_‐P.

Figure 5c depicts the cycling stability of the Bi_3_Nb_17_O_47_/Li and Bi_3_Nb_17_O_47_‐P/Li cells at 5 C. Bi_3_Nb_17_O_47_ shows unsatisfactory cyclic stability, with a small capacity retained at 38 mAh g^−1^ after 1000 cycles, corresponding to a relatively low capacity retention of 77.1%. In contrast, obviously higher capacity retention of 84.9% is obtained for Bi_3_Nb_17_O_47_‐P, due to its more stable structure with smaller unit‐cell‐volume fluctuation during the GCD processes. To further clarify the nature of the Nb migration and verify the structural stability, ex situ X‐ray photoelectron spectroscopy (XPS) analyses were performed on the electrodes before and after cycling at 0.5 C (Figure S7). The results show that the Nb 3*d* binding energies remain unchanged, indicating that the Nb cation maintains a stable +5 oxidation state without chemical reduction. This chemical stability corroborates the excellent high‐mass‐loading electrodes (8.4 mg cm^−2^) electrochemical durability observed in the 0.5 C cycling test (Figure S8), where the Bi_3_Nb_17_O_47_‐P anode retains 96.6% of its capacity. Collectively, these results confirm that the observed Nb migration corresponds to stable ferroelectric off‐centering displacements rather than irreversible chemical valence changes.

## 3 Conclusion

This study introduces a transformative ferroelectric‐polarization strategy to overcome the intrinsic limitations of TTB‐type anodes in LIBs, using Bi_3_Nb_17_O_47_ as a model system. By exploiting the non‐centrosymmetric crystal structure and field‐induced ionic displacements, we have achieved controlled structural engineering that simultaneously enhances Li^+^‐storage capability and structural stability. The polarization triggers asymmetric Nb^5+^ displacement from octahedral centers, coupled with inward O^2−^ contraction via Nb−O interactions, expanding Li^+^‐storage cavities by approximately 8%. In situ XRD and Rietveld refinements reveal that the polarization reduces unit‐cell‐volume fluctuations during cycling, stabilizing the TTB framework against Li^+^ induced strain. The diminished (002)/(001) peak ratio in Bi_3_Nb_17_O_47_‐P confirms persistent Nb^5+^ displacements along the *z*‐axis, which optimize Li^+^‐transport pathways and buffer lattice distortions. Ferroelectric characterizations validate operational stability under LIBs conditions (<80°C), with minimal polarization loss in the 20°C−100°C range. This structural optimization boosts the reversible capacities by 36%−55% across 0.1−10 C rates, with Bi_3_Nb_17_O_47_‐P delivering 242 mAh g^−1^ at 0.1 C and retaining 84.9% capacity after 1000 cycles at 5 C, significantly outperforming those in the pristine Bi_3_Nb_17_O_47_. This work pioneers a dynamic and field‐driven paradigm for electrode design, transcending conventional doping/defect approaches. The synergy between ferroelectric polarization and electrochemical performance highlights its potential for scalable implementation in LIBs and adaptability to other metal‐ion batteries.

## 4 Experimental

### 4.1 Material Preparations

Bi_3_Nb_17_O_47_ micron‐sized particles were successfully synthesized using a one‐step solid‐state reaction within the Bi_2_O_3_–Nb_2_O_5_ system at a high calcination temperature of 1170°C. Bi_2_O_3_ (Macklin, 99.9%) and Nb_2_O_5_ (Macklin, 99.9%) were mixed in a zirconia pot at a molar ratio of 3:17. Subsequently, 10 mL ethanol was introduced as a dispersing agent. The mixture was ball‐milled in a SPEX 8000 m mixer for 1 h. After drying, the powder was calcined at 1170°C for 4 h in air with a heating rate of 3°C min^−1^, obtaining the Bi_3_Nb_17_O_47_ micron‐sized particles. After the second grinding, the powder achieved a uniform particle size distribution. An organic binder, specifically 10% polyvinyl alcohol by weight, was incorporated, and the reground powder was compressed into cylindrical compacts measuring 13 mm in diameter and 1–2 mm in thickness under a pressure of about 200 MPa. Bi_3_Nb_17_O_47_ ceramics were achieved by calcination at 1170°C for 4 h.

### 4.2 Corona Discharge Poling

The schematic diagram of an in‐house designed corona discharge poling setup is presented in Figure 1a. This system primarily consists of two electrodes: (i) a sharp‐tipped conical copper needle with a diameter of approximately 5 mm, known as the corona needle, and (ii) a large, thick (20 mm) smooth copper disk. When a high direct current (DC) voltage is applied between these electrodes, the electric field intensity around the tip of the corona needle becomes extremely strong, leading to the ionization of the surrounding air. Consequently, the area around the powder sample becomes populated with positive ions and free electrons. It is important to note that the powdered sample was placed on the copper disc, and the distance between the needle tip and copper disc was maintained at 2 mm. The DC voltage was gradually increased until the desired poling field (2.4 kV mm^−1^) was achieved, and the poling process was 20 mins. After completing the poling process, the poled Bi_3_Nb_17_O_47_ sample (Bi_3_Nb_17_O_47_‐P) was removed from the copper disc and carefully collected. For comparison, the unpoled Bi_3_Nb_17_O_47_ sample was named Bi_3_Nb_17_O_47_.

### 4.3 Structure and Morphology Characterizations

The powder X‐ray diffraction (XRD) patterns of Bi_3_Nb_17_O_47_ and Bi_3_Nb_17_O_47_‐P were recorded on a Bruker D8 X‐ray diffractometer with Cu‐K*α* radiation. The diffraction data were collected in a 10°–80° range (2*θ*) at a scanning speed of 5° min^−1^. Rietveld refinements of the XRD patterns were conducted using the GSAS program to analyze the crystal structures [40]. The particle sizes, morphologies, and microstructures of the Bi_3_Nb_17_O_47_ samples were characterized by field‐emission scanning electron microscopy (FESEM, JEOL JSM‐7800F) equipped with energy dispersive X‐ray spectrometry (EDX, OXFORD X‐Max), high‐resolution transmission electron microscopy (HRTEM, JEOL JEM‐2100), and spherical aberration corrected transmission electron microscope (TitanCubed G2 60–300). Valence‐state analyses were performed via X‐ray photoelectron spectroscopy (XPS) using a PHI 5000 Versaprobe III system with a monochromatic Al‐K*α* source.

### 4.4 Dielectric and Ferroelectric Characterizations

The dielectric characteristics were measured using a dielectric spectrometer (Turnkey Concept 50, Novocontrol Technologies) across a wide temperature range (30°C–800°C) at 1 MHz with a temperature change rate of 3°C min^−1^. The polarization‐field hysteresis loops were assessed at various temperatures employing a Precision Materials Analyzer (RT Premier II, Radient Technologies).

### 4.5 Electrochemical Tests

The electrochemical properties of Bi_3_Nb_17_O_47_ and Bi_3_Nb_17_O_47_‐P were evaluated using standard 2032‐type coin cells assembled in an argon‐filled glovebox. Each half cell consisted of a working electrode (Bi_3_Nb_17_O_47_ or Bi_3_Nb_17_O_47_‐P), a lithium‐foil reference/counter electrode, and a polyethylene separator saturated with non‐aqueous electrolyte (1 m LiPF_6_ in an ethylene carbonate/diethylene carbonate/dimethyl carbonate mixed organic solvent (1:1:1 in volume)). The working electrodes with different active‐material loadings contain a dry mixture with active materials, Super‐P carbon, and polyvinylidene fluoride (8:1:1 in mass) on copper foils. The electrochemical properties and the galvanostatic intermittent titration technique (GITT) assays at 25°C were examined by galvanostatic charge–discharge (GCD) tests on a multichannel battery tester (Neware CT‐3008). Cyclic voltammetry (CV) curves and electrochemical impedance spectroscopy (EIS) were recorded in a Metrohm Autolab electrochemical workstation.

### 4.6 In‐Situ Characterizations

The assembly of in situ XRD cells for room temperature was the same as that in the half cells except that a beryllium plate served as not only the current collectors but also the low‐X‐ray‐penetration windows in commercial modules (Scistar LIBs‐XRD).

## Funding

This work was supported by the Fundamental Research Funds for the Central Universities (2232025A‐14, 2232024A‐01), the Seed Funding for the Center of Acoustic Functional Materials and Applications (No. 16002101), National Natural Science Foundation of China (Nos. 52572211, 52231007, 12327804, T2321003, 22088101, 22405050), and the National Key Research Program of China (Nos. 2024YFA1208902, 2024YFA1408000, 2021YFA1200600).

## Conflicts of Interest

The authors declare no conflicts of interest.

## Supporting information




**Supporting File**: advs74021‐sup‐0001‐SuppMat.docx.

## Data Availability

The data that support the findings of this study are available from the corresponding author upon reasonable request.
