# Supplementary material for: Ferroelectric‐Polarization‐Driven Structural Engineering of Bi3Nb17O47 Anodes for High‐Performance Lithium‐Ion Batteries
Source: Adv Sci (Weinh). 2026 Jan 22;13(19):e20293. doi: 10.1002/advs.202520293 (PMC13045299; doi:10.1002/advs.202520293)
Supplement: Supplementary file 1 — Supporting File: advs74021‐sup‐0001‐SuppMat.docx. [file ADVS-13-e20293-s001.docx]

Supporting Information

Ferroelectric-Polarization-Driven Structural Engineering of Bi_3_Nb_17_O_47_ Anodes for High-Performance Lithium-Ion Batteries

*Xiaoming Lou, Songjie Li, Chunfu Lin*, Yanchao Li, Mingze Sun, Zhen Liu, Weiye Nie, Xiaolong Tang, Baoming Wang*, Wenwu Cao* and Renchao Che**

Chunfu Lin, Renchao Che

College of Physics, Donghua University, Shanghai 201620, China

E-mails: [linchunfu@dhu.edu.cn](mailto:linchunfu@dhu.edu.cn) (C. Lin)

Xiaoming Lou, Yanchao Li, Mingze Sun, Weiye Nie, Baoming Wang, Wenwu Cao

Center of Acoustic Functional Materials and Applications, School of Materials Science and Intelligent Engineering, Nanjing University, Suzhou 215163, China

E-mails: [baoming@nju.edu.cn](mailto:baoming@nju.edu.cn) (B. Wang); [wcao@nju.edu.cn](mailto:wcao@nju.edu.cn) (W. Cao)

Renchao Che

Laboratory of Advanced Materials, Shanghai Key Lab of Molecular Catalysis and Innovative Materials, State Key Laboratory of Coatings for Advanced Equipment, College of Smart Materials and Future Energy, Fudan University, Shanghai 200438, China

E-mail: [rcche@fudan.edu.cn](mailto:rcche@fudan.edu.cn) (R. Che)

Songjie Li, Chunfu Lin

Institute of Materials for Energy and Environment, School of Materials Science and Engineering, Qingdao University, Qingdao 266071, China

Zhen Liu, Xiaolong Tang

School of Materials Science and Engineering, Nanjing University of Science and Technology, Nanjing 210094, Jiangsu, China


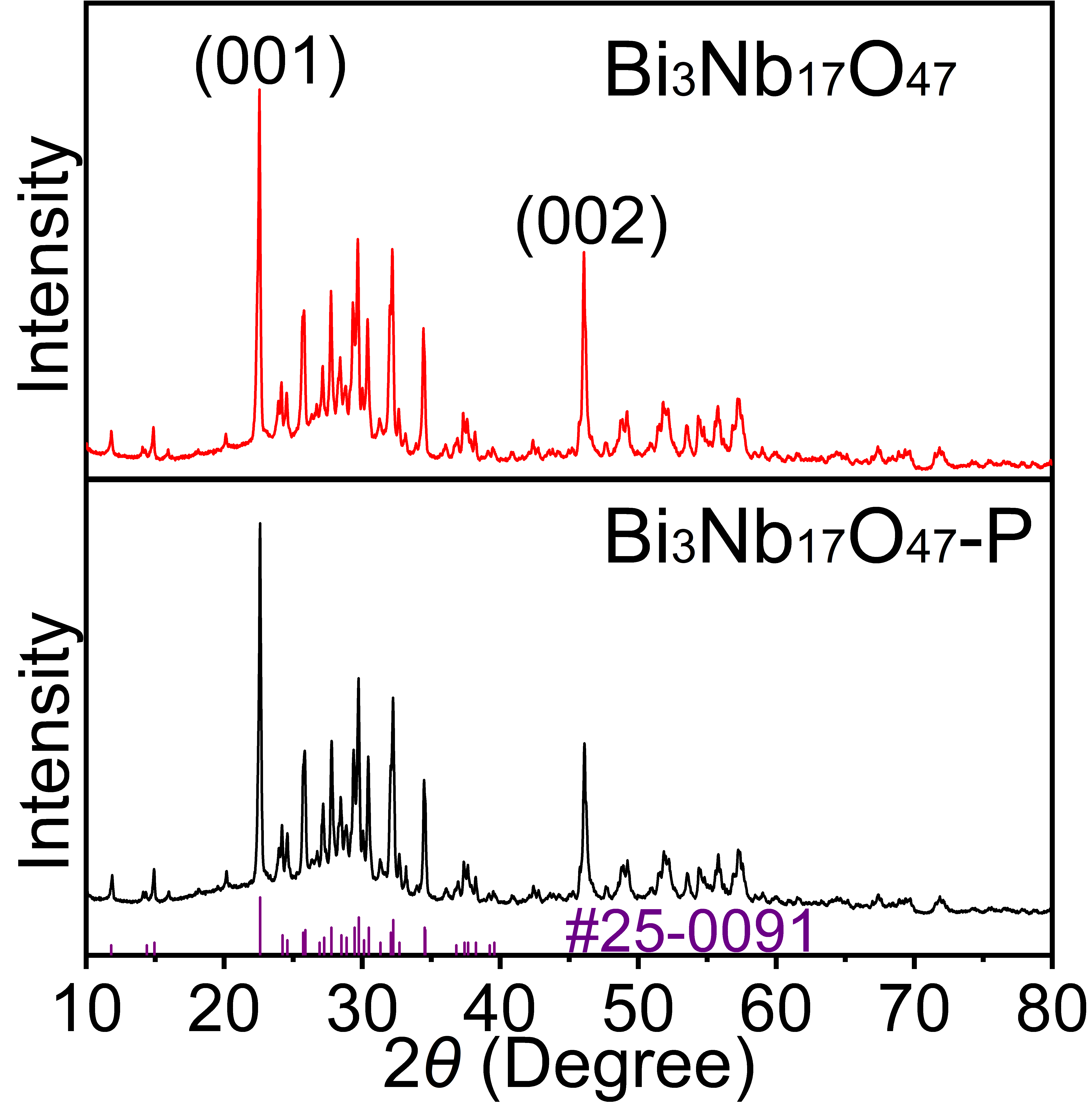


**Figure S1.** XRD patterns of Bi_3_Nb_17_O_47_ and Bi_3_Nb_17_O_47_-P powders.


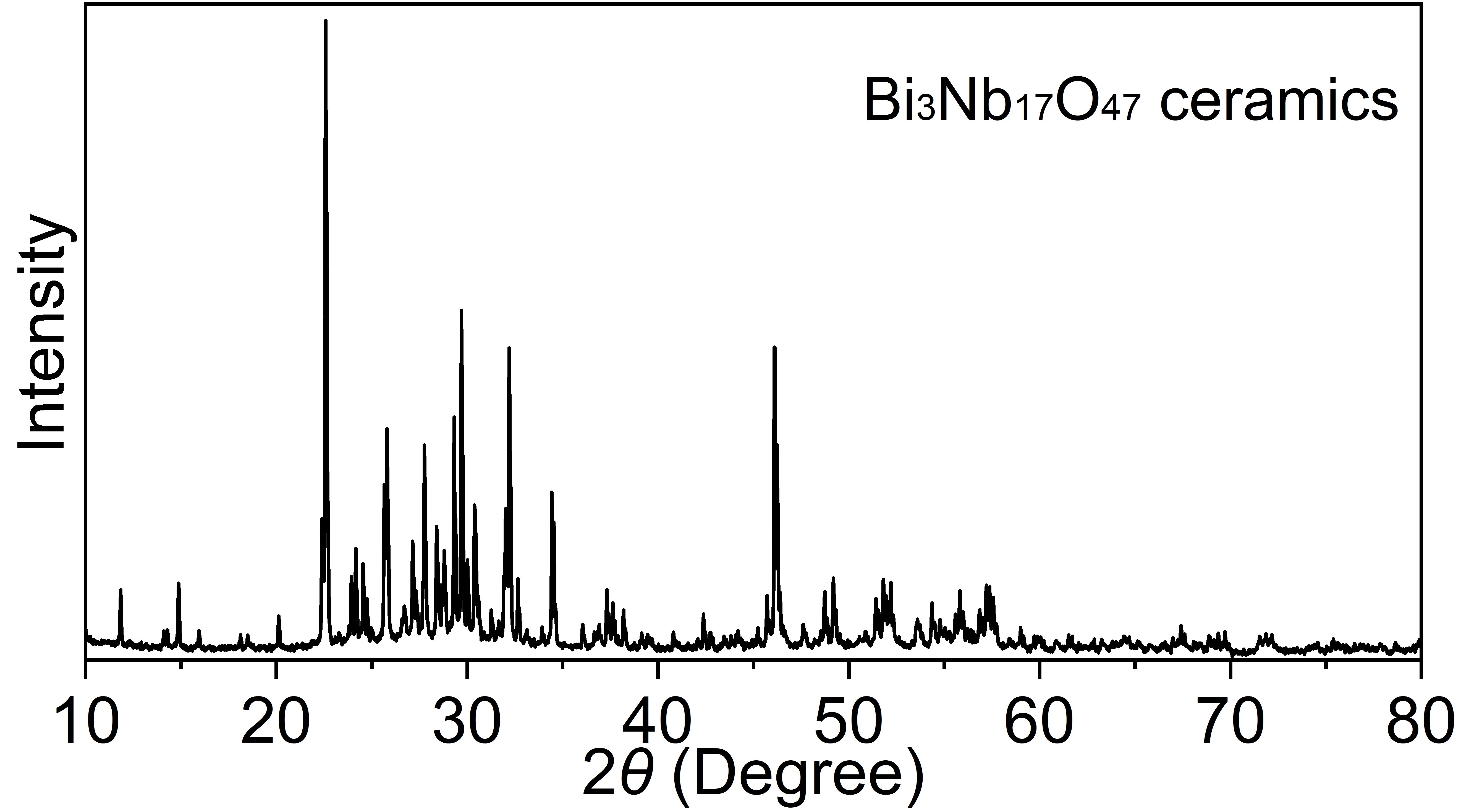


**Figure S2.** XRD pattern of Bi_3_Nb_17_O_47_ ceramics.


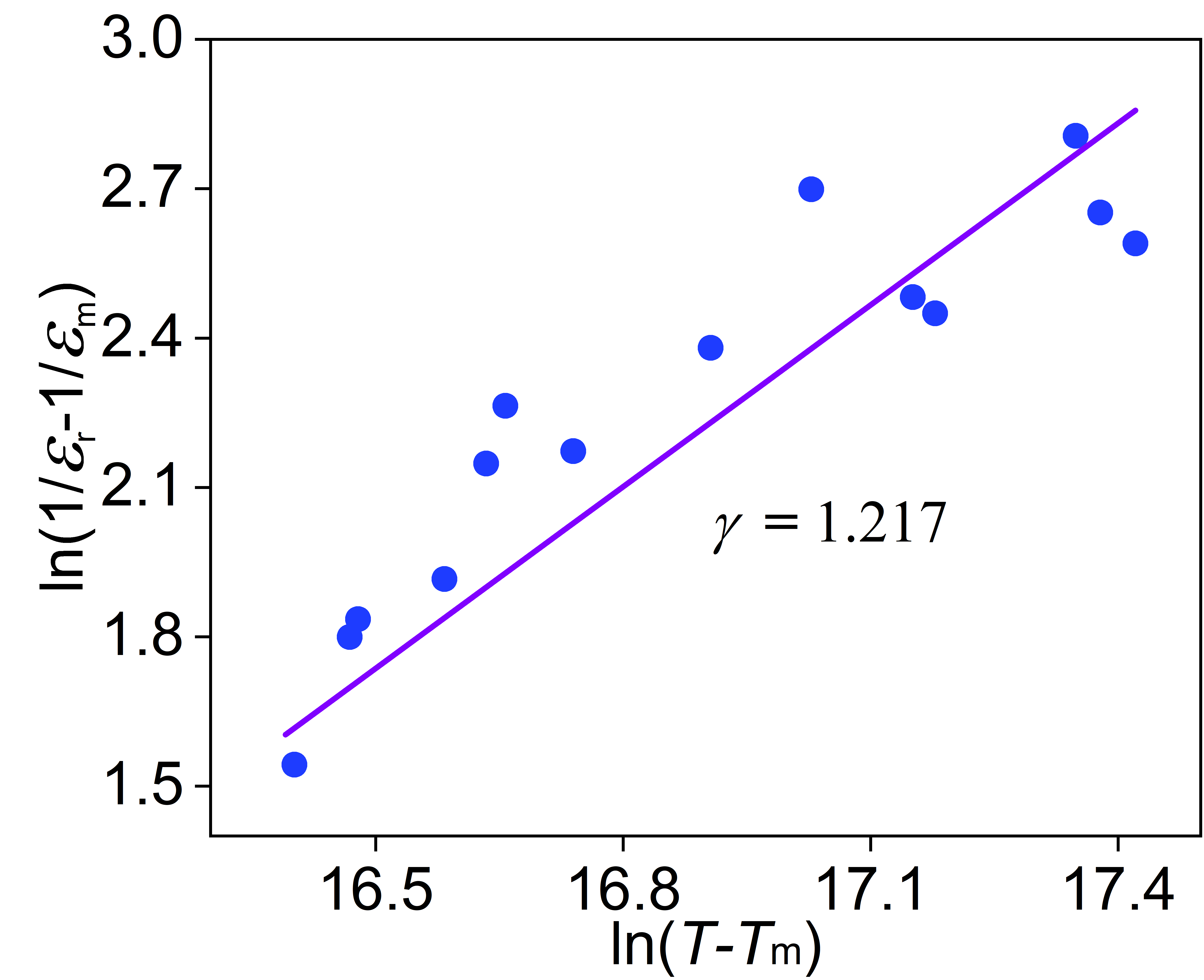


**Figure S3.** Relaxation characteristic of Bi_3_Nb_17_O_47_.


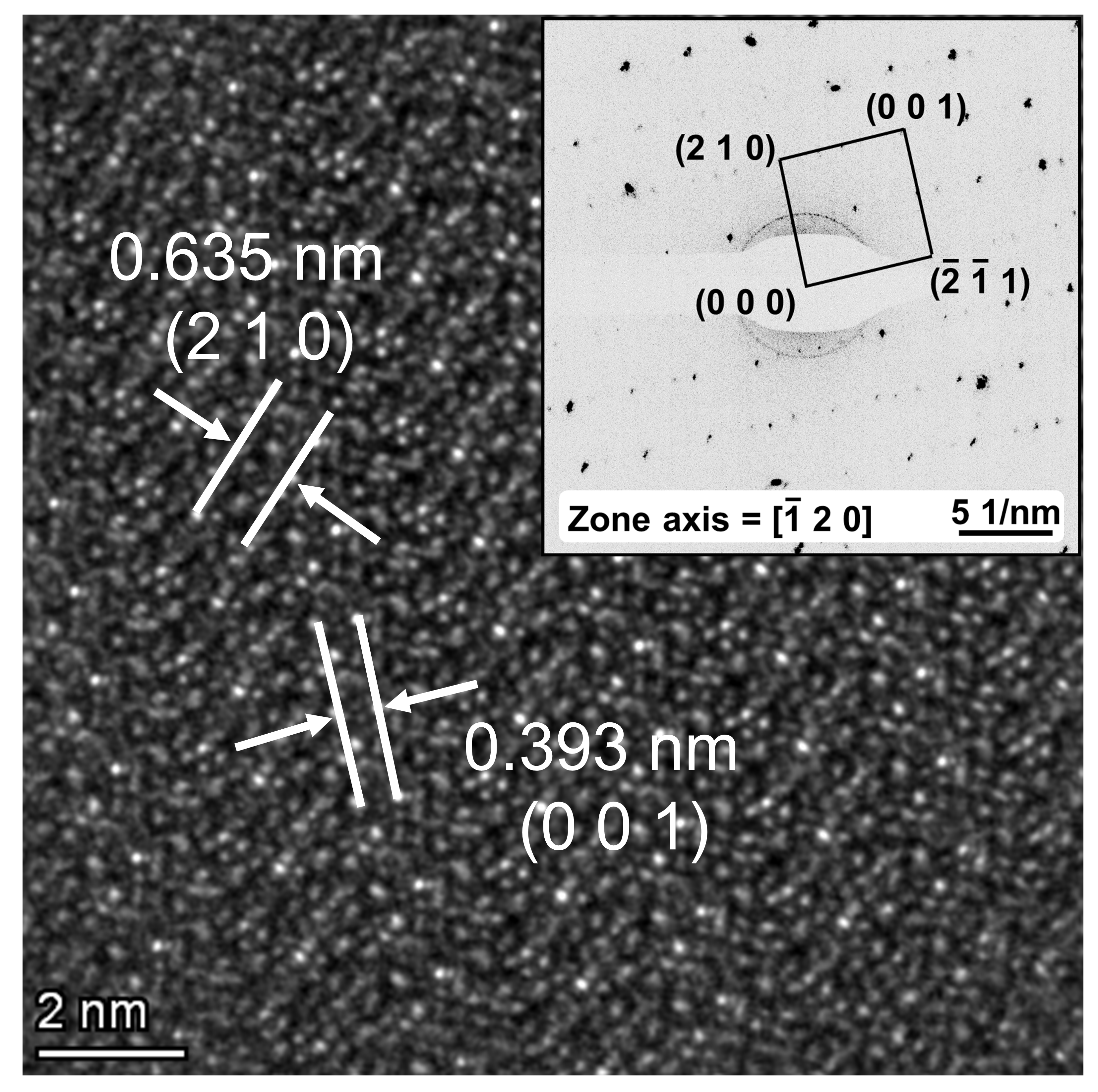


**Figure S4.** HRTEM image of Bi_3_Nb_17_O_47_ (*inset*: SAED pattern).


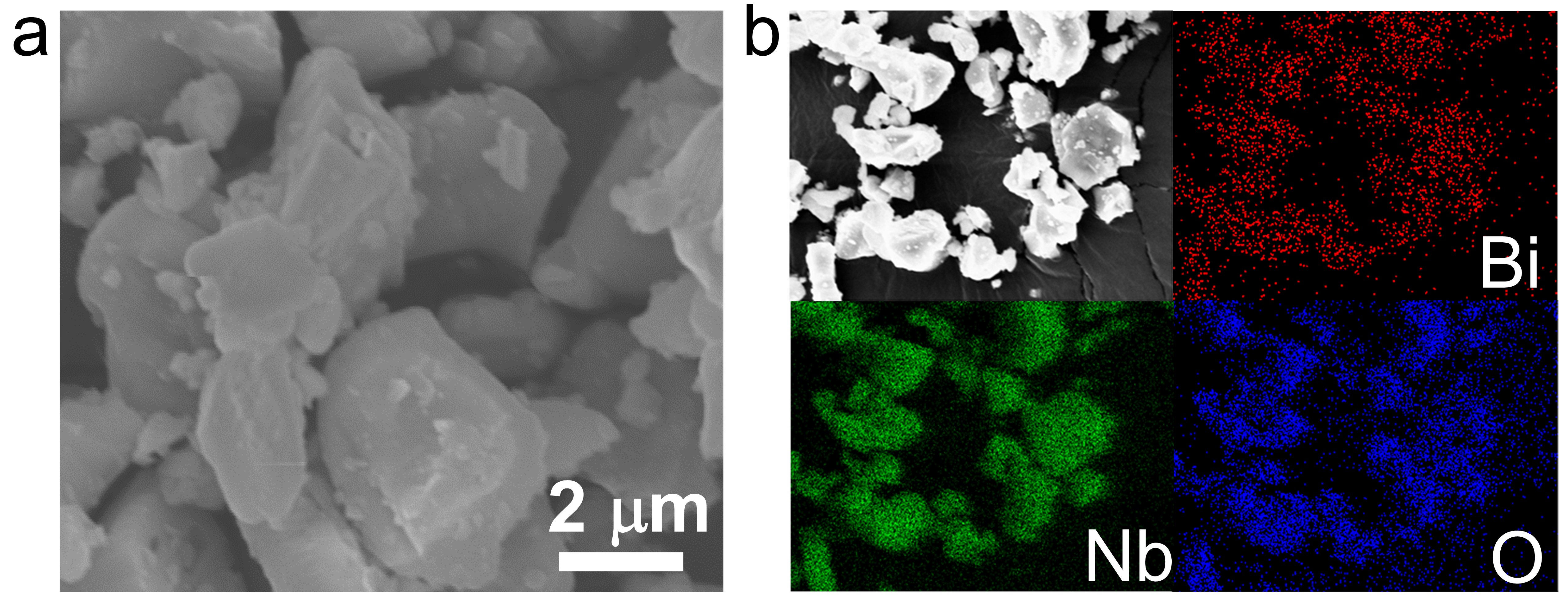


**Figure S5.** a) FESEM image and b) EDX mapping images of Bi_3_Nb_17_O_47_.


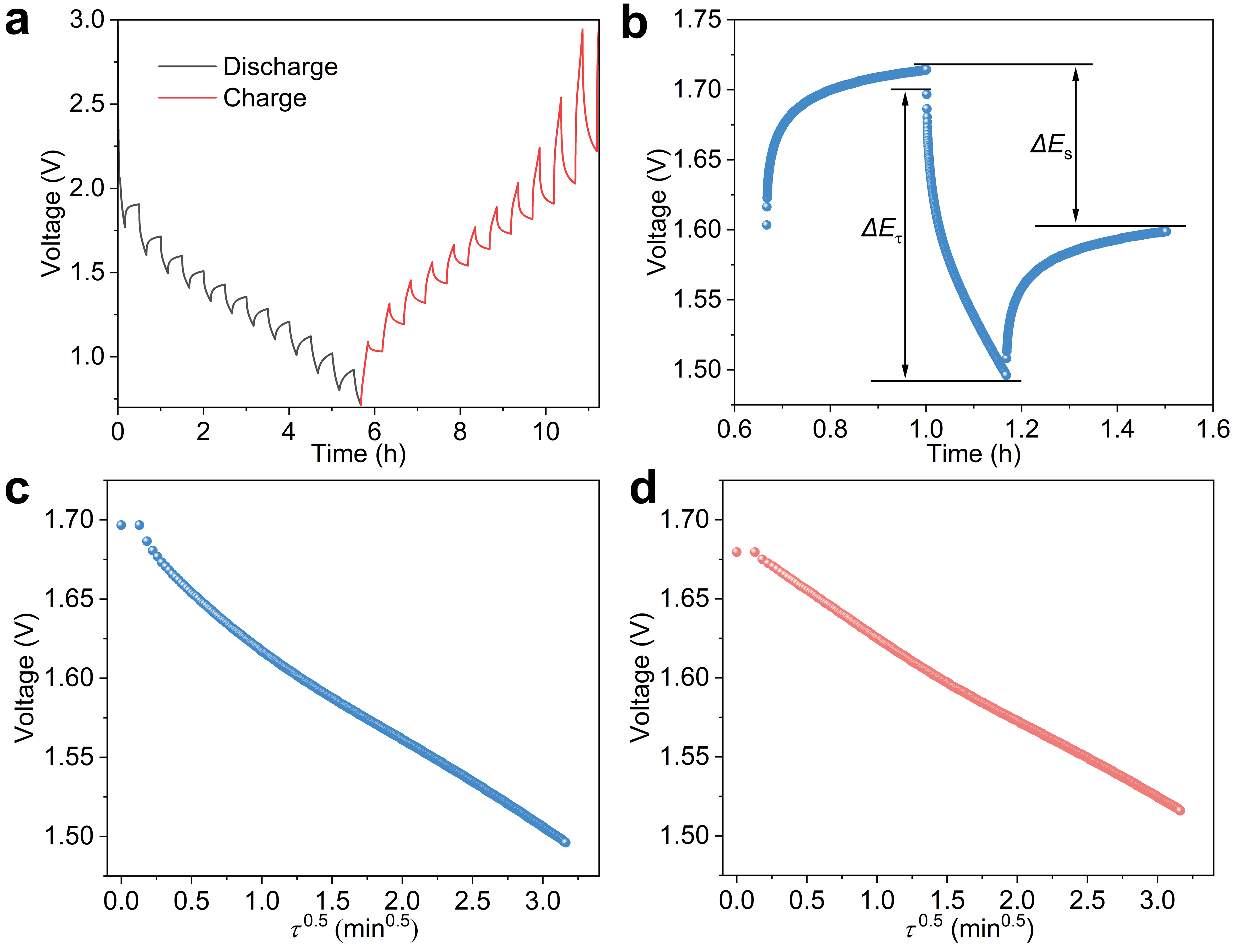


**Figure S6.** a) Second-cycle GITT discharge‒charge profiles of Bi_3_Nb_17_O_47_ at 0.5C. b) *E* *vs.* *t* profiles for a single step in GITT experiment of Bi_3_Nb_17_O_47_ at 0.5 C. Linear behavior of *E* *vs.* *τ*^0.5^ relationship during a typical titration in c) Bi_3_Nb_17_O_47_ and d) Bi_3_Nb_17_O_47_-P.

**Calculations of apparent Li^+^-diffusion coefficients from GITT**

The GITT tests were conducted on the two half cells with a 10-minute pulse current at 0.5 C between 20-minute rest intervals to study the Li^+^ diffusivity in Bi_3_Nb_17_O_47_ and Bi_3_Nb_17_O_47_-P. **Figure** **4c** and **Figure S6a** respectively exhibit the typical GITT curves of the Bi_3_Nb_17_O_47_/Li and Bi_3_Nb_17_O_47_-P/Li half cells during the second lithiation–delithiation cycle. For a clear observation, a single step of GITT is presented in **Figure** **4d** and **Figure S6b**. Based on the Fick’s second law, the apparent Li^+^-diffusion coefficients *D*_Li_ of Bi_3_Nb_17_O_47_/Bi_3_Nb_17_O_47_-P can be calculated by using **Equation S1**:

$D_{\mathrm{Li}}= \frac{4}{\pi}{(\frac{m_{b}V_{m}}{M_{b}S})}^{2}{(\frac{E_{s}}{E/d\sqrt{}})}^{2} (\ll\frac{L^{2}}{D_{\mathrm{Li}}})$ (S1)

where $M_{b}$ is the molar mass of Bi_3_Nb_17_O_47_/Bi_3_Nb_17_O_47_-P, $V_{m}$ is the molar volume of Bi_3_Nb_17_O_47_/Bi_3_Nb_17_O_47_-P, $m_{b}$ is the mass of Bi_3_Nb_17_O_47_/Bi_3_Nb_17_O_47_-P, $S$ is the Bi_3_Nb_17_O_47_/Bi_3_Nb_17_O_47_-P electrode area, $\tau$ is the pulse duration time, $L$ is the Bi_3_Nb_17_O_47_/Bi_3_Nb_17_O_47_-P electrode thickness, and $\Delta E_{s}$ and $\Delta E_{\tau}$ respectively represent the change in the equilibrium potential and the change in potential during the current pulse, which can be gained from the GITT curves (**Figure** **4d** and **Figure S6b**). As the potential during a single titration delivers a linear relationship with $\tau^{0.5}$ (**Figure S6c**,**d**), **Equation S1** can be simplified as **Equation S2**:

$D_{\mathrm{Li}}= \frac{4}{\pi\tau}{(\frac{m_{b}V_{m}}{M_{b}S})}^{2}{(\frac{E_{s}}{E})}^{2} (\ll\frac{L^{2}}{D_{\mathrm{Li}}})$ (S2)

Based on **Equation S2**, the apparent Li^+^-diffusion coefficients during different states of discharge/charge are obtained, and displayed in **Figure 4f**.


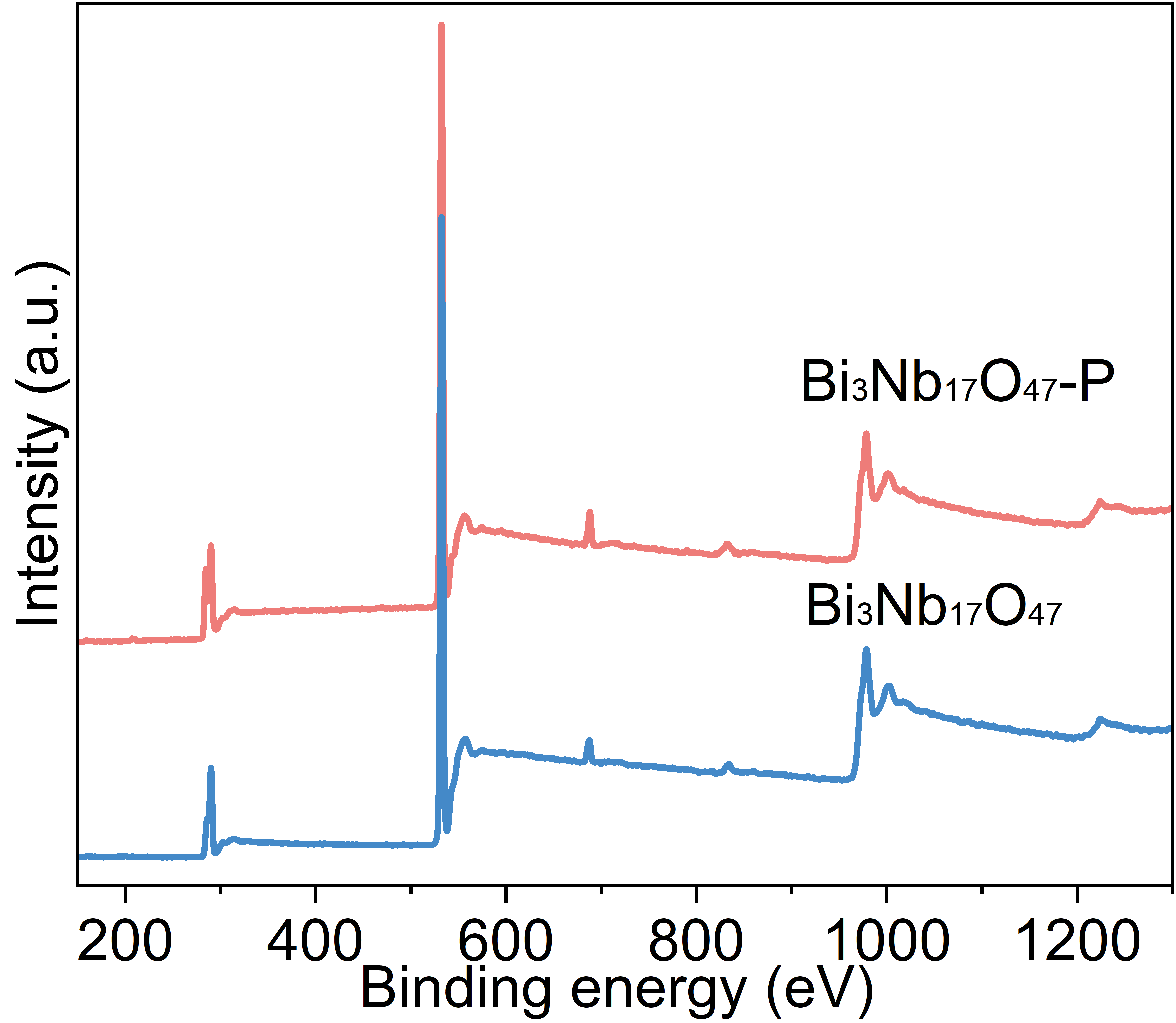


**Figure S7.** *Ex-situ* XPS spectra of Bi_3_Nb_17_O_47_ and Bi_3_Nb_17_O_47_-P at 3.0 V.


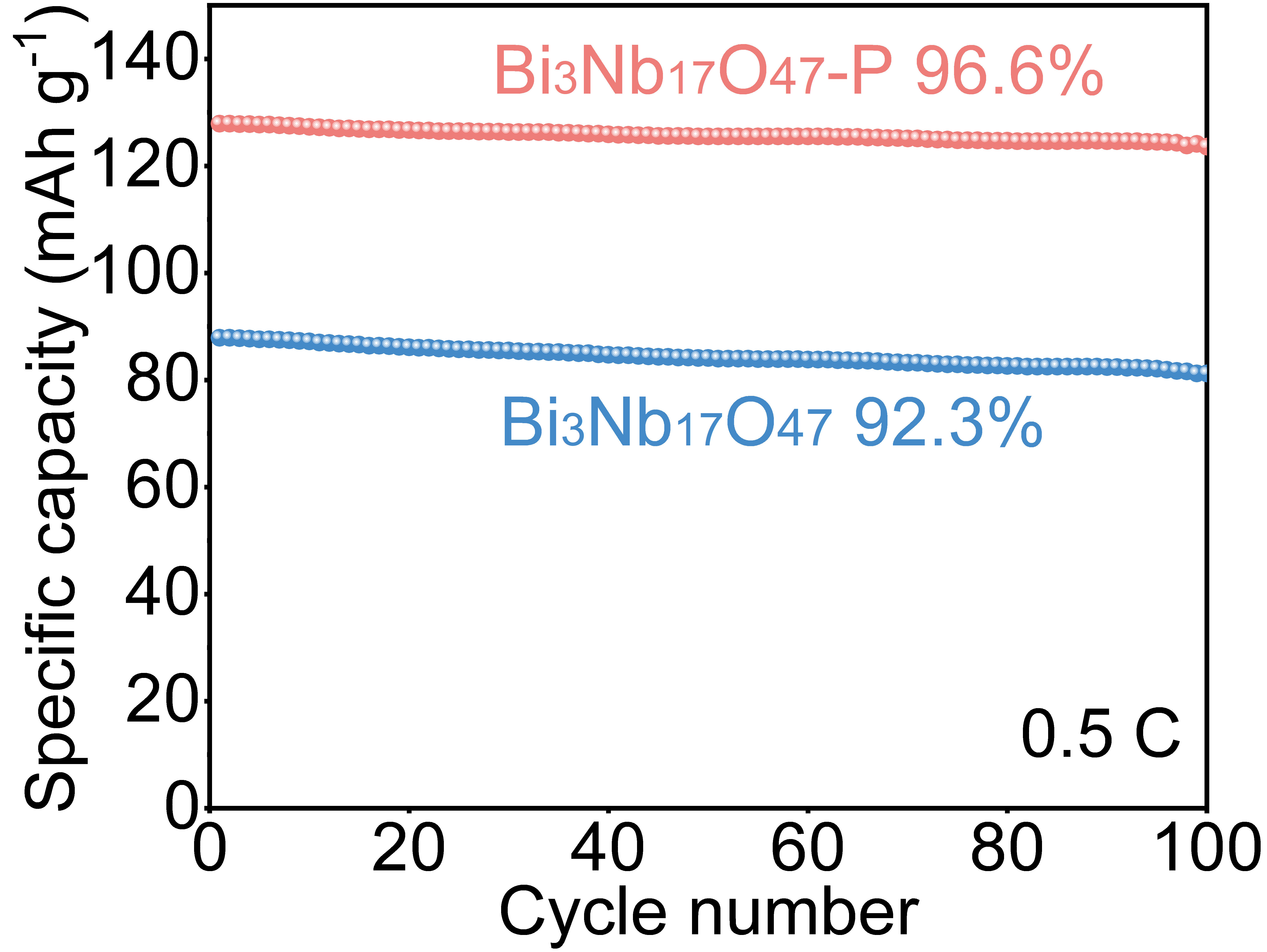


**Figure S8.** Cycling stability of Bi_3_Nb_17_O_47_/Li and Bi_3_Nb_17_O_47_-P/Li half cells at 0.5 C (mass loading: 10.5 mg cm^−2^ ).

**Table S1.** Atomic parameters of Bi_3_Nb_17_O_47_ and Bi_3_Nb_17_O_47_-P (space group: *Pba*2).

| atom | *x* | *y* | *z* | occupancy | thermal displacement |
| --- | --- | --- | --- | --- | --- |
| Bi1 | 0.7446/0.7476 | 0.2900/0.2920 | 0.0340/0.0399 | 0.5/0.5 | 0.0717/0.0571 |
| Bi2 | 0.3716/0.3715 | 0.0577/0.0577 | 0.0704/0.0706 | 0.5/0.5 | 0.1829/0.1785 |
| Bi3 | 0.9758/0.9889 | 0.0205/0.0176 | 0.1215/0.1205 | 0.5/0.5 | 0.4371/0.3656 |
| Nb1 | 0.3384/0.3441 | 0.3921/0.3902 | 0.5139/0.5291 | 1/1 | 0.0774/0.0588 |
| Nb2 | 0.0712/0.0738 | 0.0698/0.0719 | 0.6087/0.6320 | 1/1 | 0.0323/0.0261 |
| Nb3 | 0.0770/0.0769 | 0.4042/0.4022 | 0.5961/0.5986 | 1/1 | 0.0680/0.0624 |
| Nb4 | 0.4489/0.4468 | 0.2323/0.2334 | 0.5102/0.5481 | 1/1 | 0.0557/0.0421 |
| Nb5 | 0.2999/0.2999 | 0.4782/0.4783 | 0.5398/0.5826 | 1/1 | 0.0121/0.0201 |
| Nb6 | 0.2306/0.2235 | 0.3088/0.3085 | 0.5313/0.5593 | 1/1 | 0.0447/0.0599 |
| Nb7 | 0.2915/0.2949 | 0.1352/0.1362 | 0.5081/0.5180 | 1/1 | 0.0355/0.0390 |
| Nb8 | 0.0160/0.0200 | 0.1720/0.1702 | 0.5694/0.5990 | 1/1 | 0.0915/0.1326 |
| Nb9 | 0.5/0.5 | 0/0 | 0.5964/0.5263 | 1/1 | 0.0127/0.0289 |
| O1 | 0.3554/0.3551 | 0.9969/0.9968 | 0.2531/0.2525 | 1/1 | 0.0556/0.0558 |
| O2 | 0.1336/0.1325 | 0.0321/0.0315 | 0.2678/0.2668 | 1/1 | 0.0161/0.0211 |
| O3 | 0.4655/0.4605 | 0.0515/0.0418 | 0.5404/0.5404 | 1/1 | 0.5821/0.5121 |
| O4 | 0.4655/0.4155 | 0.1286/0.1081 | 0.6996/0.6985 | 1/1 | 0.01/0.01 |
| O5 | 0.2547/0.2544 | 0.1002/0.0915 | 0.5015/0.5056 | 1/1 | 0.0055/0.0051 |
| O6 | 0.9819/0.9816 | 0.1189/0.1187 | 0.6015/0.6012 | 1/1 | 0.01/0.01 |
| O7 | 0.1273/0.1354 | 0.1491/0.1616 | 0.9134/0.9112 | 1/1 | 0.0976/0.0842 |
| O8 | 0.3535/0.3531 | 0.1705/0.1704 | 0.5102/0.5104 | 1/1 | 0.0289/0.0324 |
| O9 | 0.2678/0.2673 | 0.2476/0.2473 | 0.9658/0.9656 | 1/1 | 0.0584/0.0441 |
| O10 | 0.0357/0.0343 | 0.2899/0.2896 | 0.6403/0.6403 | 1/1 | 0.01/0.01 |
| O11 | 0.0386/0.0376 | 0.2265/0.2136 | 0.4421/0.4457 | 1/1 | 0.0143/0.0116 |
| O12 | 0.3694/0.3586 | 0.3315/0.3324 | 0.5896/0.5895 | 1/1 | 0.01/0.01 |

**Table S1.** (continued).

| atom | *x* | *y* | *z* | occupancy | thermal displacement | |
| --- | --- | --- | --- | --- | --- | --- |
| O13 | 0.1363/0.1363 | 0.3677/0.3677 | 0.5611/0.5625 | 1/1 | | 0.7041/0.6949 |
| O14 | 0.2164/0.2031 | 0.4155/0.4233 | 0.4641/0.4636 | 1/1 | | 0.01/0.01 |
| O15 | 0.4207/0.4288 | 0.4340/0.4327 | 0.5843/0.5796 | 1/1 | | 0.01/0.01 |
| O16 | 0.3584/0.3579 | 0.3943/0.3945 | 0.9919/0.9920 | 1/1 | | 0.01/0.01 |
| O17 | 0.0575/0.0572 | 0.0754/0.0743 | 0.1415/0.1424 | 1/1 | | 0.0668/0.0717 |
| O18 | 0.0481/0.0475 | 0.3950/0.3936 | 0.9542/0.9547 | 1/1 | | 0.0578/0.0543 |
| O19 | 0.4581/0.4581 | 0.2333/0.2328 | 0.0753/0.0754 | 1/1 | | 0.2692/0.1994 |
| O20 | 0.2405/0.2412 | 0.4804/0.4899 | 0.1255/0.1258 | 1/1 | | 0.01/0.01 |
| O21 | 0.3030/0.3035 | 0.1229/0.1212 | 0.0743/0.0736 | 1/1 | | 0.0229/0.0227 |
| O22 | 0.0095/0.0091 | 0.1706/0.1704 | 0.0693/0.0691 | 1/1 | | 0.0253/0.0212 |
| O23 | 0.5/0.5 | 0/0 | 0.1389/0.1324 | 1/1 | | 0.01/0.01 |
| O24 | 0.1898/0.1884 | 0.3139/0.3126 | 0.8927/0.8987 | 1/1 | | 0.0223/0.0208 |

**Table S2.** Rietveld-refined lattice parameters of Bi_3_Nb_17_O_47_ and Bi_3_Nb_17_O_47_-P.

| sample | space group | *a* (Å) | *b* (Å) | *c* (Å) | *V* (Å^3^) | *^*^R*_wp_ |
| --- | --- | --- | --- | --- | --- | --- |
| Bi_3_Nb_17_O_47_ | *Pba*2 | 12.528 | 37.11 | 3.93 | 1827.95 | 0.053 |
| Bi_3_Nb_17_O_47_-P | *Pba*2 | 12.534 | 37.13 | 3.93 | 1830.96 | 0.043 |

^*^*R*_wp_: weighted profile residual.
